# Emerging Invasive Weeds in Iran: Occurrence, Ecological Impacts, and Sustainable Management

**DOI:** 10.3390/plants14172611

**Published:** 2025-08-22

**Authors:** Ali Reza Yousefi, Sirwan Babaei, Iraj Nosratti, Ehsan Zeidali, Masoumeh Babaei, Ebrahim Asadi Oskouei, Hesan Saberi, Mandeep Redhu, Amir Sadeghpour

**Affiliations:** 1 Department of Plant Production and Genetics, University of Zanjan, Zanjan 45371-38791, Iran; 2 Department of Plant Production and Genetics, University of Kurdistan, Sanandaj 66177-15175, Iran; 3 Department of Plant, Soil and Agricultural System, Southern Illinois University, Carbondale, IL 62901, USA; 4 Department of Plant Production & Genetics, The Campus of Agriculture and Natural Resources, Razi University, Kermanshah 67144-14971, Iran; 5 Department of Agronomy and Plant Breeding, Faculty of Agriculture, Ilam University, Ilam 77111-69391, Iran; 6 Department of Biological Sciences, University of Kurdistan, Sanandaj 66177-15175, Iran; 7 Research Institute of Meteorology and Atmospheric Science, Tehran 13611-14977, Iran

**Keywords:** invasive weeds, biological invasions, global biodiversity, ecological impact, agricultural weeds, weed management

## Abstract

Invasive weeds pose a growing threat to global biodiversity, ecosystem stability, and agricultural productivity with significant ecological and economic consequences. In Iran, the rapid spread of invasive species such as *Boreava orientalis*, *Azolla* spp., *Ibicella lutea*, *Physalis divaricata*, *Picnomon acarna*, *Cynanchum acutum*, *Vicia hyrcanica*, *Eichhornia crassipes*, and *Ambrosia psilostachya* has severely affected native ecosystems, disrupted ecological processes, and threatened food security. These species exhibit aggressive traits such as rapid maturity, high reproductive rates, seed dormancy, and allelopathy that enable them to outcompete native species and successfully invade and dominate delicate habitats. Despite their documented impacts, a critical gap remains in understanding their biology, ecology, and management, particularly in understudied regions like Iran. This review synthesizes current knowledge on major invasive weeds affecting Iranian agroecosystems, with a focus on their ecological impacts and the urgent need for sustainable management strategies. It presents an integrated framework that combines ecological, biological, and management perspectives to address invasiveness, particularly in highly adaptable species like *B. orientalis* and *A. psilostachya*. This review highlights the critical role of interdisciplinary collaboration, advanced technology, and community involvement in developing effective strategies. It offers practical guidance for researchers, policymakers, and agricultural stakeholders, serving as a model for managing invasive species in other vulnerable regions. Ultimately, it supports global efforts to safeguard biodiversity, improve crop productivity, and strengthen ecological resilience against the growing threat of invasive species.

## 1. Introduction

Invasive plants and weeds pose serious ecological and economic challenges [1]; however, invasive species are particularly concerning due to their ability to spread rapidly. These species can establish in new regions, sometimes even without human intervention, often leading to substantial environmental, economic, and health impacts [2,3]. In recent years, their rapid and unregulated expansion has posed a critical threat to global ecosystem diversity, structure, and functionality [4,5]. Recent projections suggest that climate change, global transport, and socio-economic shifts will be key drivers of future alien plant invasions, intensifying their impact on native biodiversity even in the most suitable environments [6,7]. From 1980 to 2019, economic losses due to biological invasions were found to be similar to those from natural hazards, for instance, USD 1208.0 billion compared to USD 1913.6 billion from storms and USD 1139.4 billion from earthquakes [8].

Iran’s unique geographical position and diverse topography give rise to a wide range of climatic conditions, from arid deserts to lush forests, making it a hotspot for biodiversity. This rich ecological variation supports over 8000 plant species. According to recent estimates, 311 alien plant species have been recorded in Iran, among which 13 are currently classified as invasive [9]. While some of these species are fully naturalized, others possess invasive traits and may pose future ecological risks. This underscores the urgent need for systematic monitoring and proactive management. Additionally, only about 200 to 250 plant species (less than 0.3%) are considered weeds in Iranian agroecosystems, reflecting both the selectivity of weed classification and the growing concern over emerging invasives with harmful ecological and economic impacts [10,11]. The ecosystems are increasingly threatened by the introduction and spread of invasive plant species, which can outcompete native flora and disrupt ecological balances [12]. The lack of stringent biosecurity measures has further exacerbated this issue, allowing invasive species to establish and spread across various regions. Considering Iran’s distinct agricultural, environmental, and economic conditions, understanding the traits driving the success of invasive weeds is crucial. Therefore, this review article aims to fill existing knowledge gaps by identifying key traits that promote invasiveness in Iran’s ecosystems, evaluating the ecological and economic impacts on biodiversity and agriculture, and exploring sustainable management strategies to mitigate their establishment and spread.

Rather than attempting to catalog all invasive alien plant species in Iran, this review focuses on the nine most problematic and emerging species identified through national surveys, peer-reviewed literature, and expert consultations, allowing for a more detailed analysis of their classification, origin, life forms, ecological and economic impacts, and management strategies. This review is organized thematically to reflect the ecological complexity and management relevance of emerging invasive weeds in Iran. We first provide a general overview of invasive weed threats and their introduction pathways. Subsequent sections explore the ecological, economic, and agricultural impacts of these species, followed by detailed profiles of specific species. Management challenges and current control strategies are then discussed, culminating in a section on sustainable and integrated solutions. This structure is designed to move from a broad ecological context to specific species and ultimately toward practical recommendations.

## 2. Concept and Criteria of Weed Invasiveness

Weed invasiveness refers to the ability of certain plant species to establish, spread, and persist in new environments, often resulting in significant ecological or economic consequences. Unlike general weediness, which typically denotes nuisance plants in crop production, invasiveness specifically involves aggressive colonization beyond a species’ native range. Invasive plants characteristically possess traits such as rapid growth, high reproductive capacity, efficient dispersal, broad ecological tolerance, and competitive ability, often aided by reduced biotic pressure due to the absence of natural enemies like herbivores and pathogens. They often exhibit high adaptability and competitive ability, enabling them to establish, thrive, and outcompete native flora. This advantage is often linked to evolutionary trade-offs, where reduced herbivore pressure enables plants to invest less in defense mechanisms and more in growth and competition [13].

Most invasive plant species belong to a few families such as *Fabaceae*, *Asteraceae*, *Cyperaceae*, *Poaceae*, and *Mimosaceae*. For example, Mitchell and Power (2003) examined 473 European plant species that had naturalized in the United States; they found that these species faced 84% fewer fungal pathogens and 24% fewer viral pathogens in their introduced ranges compared to their native habitats, illustrating how reduced biotic stress can favor invasiveness [14]. Westbrooks (1998) identified 12 key traits commonly found in invasive weeds that enable them to thrive in new ecosystems, many of which overlap with the general attributes described above [9]. These include the rapid maturity, long-term viability, and dormancy of propagules, compatibility with dispersal vectors such as contaminated crop seed, allelopathy, physical deterrents like thorns, parasitism, mimicry of crop seeds, nutrient storage in vegetative tissues for stress tolerance, and high photosynthetic efficiency even in resource-limited environments [9].

Despite these insights, predicting invasiveness remains challenging due to an incomplete understanding of the ecological and evolutionary mechanisms underlying plant invasions [15]. While a species’ behavior in its native range is often considered, it is not a reliable predictor of its invasive potential in new regions [16]. Thus, a consistent and predictive framework for assessing invasiveness risk is still lacking.

Invasive species disrupt biogeographic boundaries and negatively impact ecosystems by reducing native species richness and abundance, increasing extinction risks, altering the genetic structure of native flora, and affecting animal behavior and trophic interactions [4]. Their ecological consequences include a drastic decline in biodiversity, loss of endangered species and their habitats, and reduced availability of suitable habitats for native insects, birds, and wildlife. Invasive plants also diminish food resources for fauna and interfere with essential ecological processes such as plant succession and disturbance regimes, including the intensity and frequency of wildfires. Furthermore, they disrupt key animal–plant interactions, including pollination, seed dispersal, and host-specific relationships, ultimately threatening ecological balance and agricultural sustainability [17].

Additionally, invasive species can alter ecosystem functioning by impacting nutrient cycling, contaminant flow, hydrological patterns, and habitat structure, thereby shifting natural ecosystem dynamics [4]. Despite these impacts, predicting which species will become invasive, and under what environmental or management conditions, remains a major challenge in invasion ecology, particularly in rapidly changing agroecosystems.

In summary, the invasive weed species discussed in this review share a suite of biological and ecological characteristics that enhance their success in new environments. These include rapid growth, early maturity, high reproductive capacity (both sexual and vegetative), seed dormancy and persistence in soil seed banks, phenotypic plasticity, allelopathy, and efficient dispersal mechanisms. Many of these species also benefit from reduced biotic pressure in their introduced ranges, such as escaping natural enemies.

Taxonomically, they span a range of families known for invasiveness, particularly Asteraceae (e.g., *Ambrosia psilostachya*), Brassicaceae (e.g., *Boreava orientalis*), Solanaceae (e.g., *Physalis divaricata*), Fabaceae (e.g., *Vicia hyrcanica*), as well as aquatic groups like Pontederiaceae and Salviniaceae (e.g., *Eichhornia crassipes*, *Azolla* spp.), and Martyniaceae (*Ibicella lutea*). These families are globally recognized for producing species that are highly adaptable, competitive, and ecologically disruptive, particularly in disturbed or resource-rich habitats.

## 3. Detailed Species Profiles

To enable a targeted and comparative assessment of major invasive weed species in Iran, taxa have been categorized into two groups according to their dominant ecological habitats and principal impact domains (Table 1). This classification facilitates a habitat-specific evaluation of the invasion dynamics and associated ecological consequences.

### 3.1. Agricultural Field Invasive Weeds

#### 3.1.1. *Ambrosia psilostachya* DC

**Figure 1:**
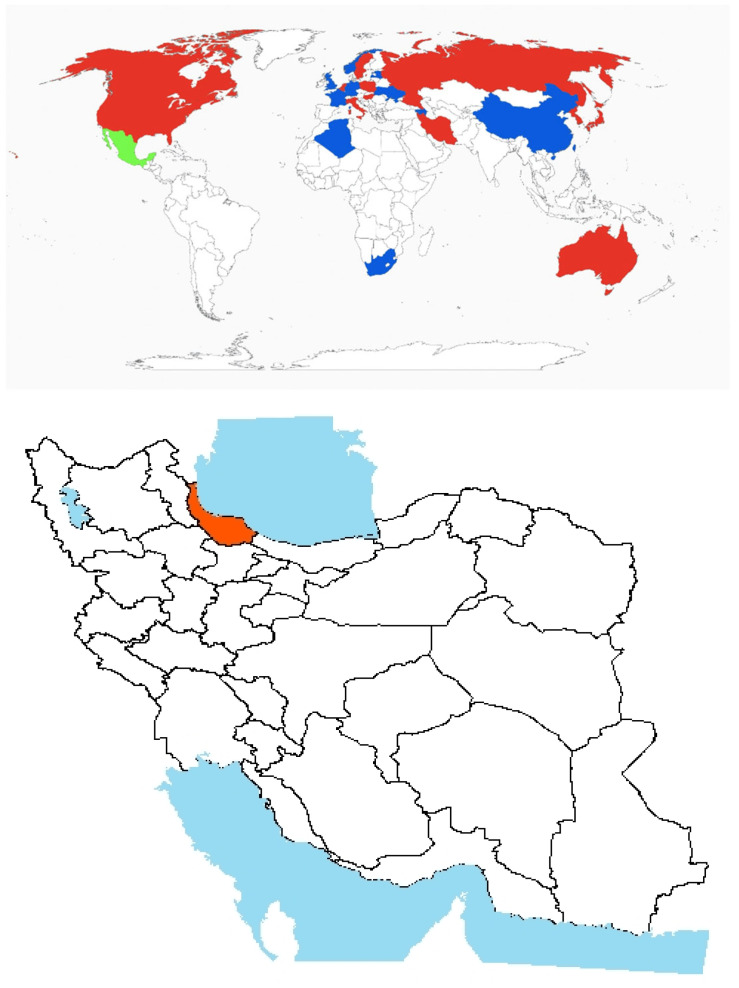
Global and national distribution of *Ambrosia psilostachya* DC. **Top panel**: Global distribution map showing native (green), invasive (red), and introduced (blue) ranges based on the literature and GBIF records (GBIF Backbone Taxonomy. Checklist dataset https://doi.org/10.15468/39omei accessed via GBIF.org on 20 August 2025). **Bottom panel**: Occurrence invasive (red) in Iran based on herbarium records, published studies, and recent field observations by the authors. Note: Absence of occurrence in some regions may reflect data gaps or lack of confirmed records, not necessarily ecological unsuitability.

- Origin and global invasiveness: The genus *Ambrosia* includes approximately 40 species and numerous subspecies, many of which are known for their allergenic properties [7]. *A. psilostachya* DC. was first recorded in Europe in the 1800s [18] and has since expanded its distribution to include North America, Europe, Asia, and other parts of the Americas [19]. It is an herbaceous perennial plant native to Mexico, where it plays a role in local ecosystems. It disrupts native biodiversity and agricultural productivity across the United States, Canada, Australia, Russia, and parts of Europe. Its presence has also been documented in China, France, Germany, and South Africa, reflecting its widespread introduction across Europe, Asia, and Africa (Figure 1) [7]. In Iran, the species was initially identified in 1991 in Anzali, Guilan Province (Figure 1). Subsequent investigations by the Guilan Research Institute confirmed its establishment in the region [20]. Since its first detection, the species has spread to several surrounding areas, including Rezvanshahr, Talesh, Astara counties, and the Rasht Trench (Figure 1).
- Morphological description, biology, and ecology: *A. psilostachya* is an erect herbaceous perennial characterized by horizontal running rootstocks, allowing it to propagate both vegetatively via rhizomes and sexually through seed production. The stems are rigidly upright and may be unbranched or heavily branched in the upper parts of the plant. Leaves are thick, hairy, and oval-lanceolate in outline, typically reaching lengths of up to 5 inches and widths of 2 inches. They are deeply dissected into narrow lobes, often with secondary lobing. The leaf surface, covered in fine hairs, tends to accumulate dust, which can hinder herbicide adhesion and reduce control efficacy [21,22]. The inflorescence is composed of unisexual flower heads, with male (staminate) and female flowers located on different parts of the same plant. Staminate flowers are small, bead-like, yellow to greenish, and short-stalked, initially densely clustered and later spreading during maturation. Pistillate (female) flowers are solitary and sparse, typically found at the base of the floral cluster, along the stem, or in axils, accompanied by large leaf-like bracts [23].

*A. psilostachya* exhibits ecological plasticity, readily colonizing a wide range of habitats, including coastal areas, dunes, sandy soils [24], riversides, and disturbed ruderal environments [25]. Although detailed climatic data for its spread are limited [23], this species thrives well under macroclimatic conditions that are conducive to coastal zones, characterized by relatively narrow diurnal and seasonal temperature fluctuations, increased precipitation during the wettest months, and consistently warmer ambient temperatures [24].

- Agricultural impact and management strategies: *A. psilostachya* poses considerable ecological and agricultural challenges due to its allelopathic properties and prolific spread along roadsides and non-agricultural lands [26]. The species releases toxic compounds that inhibit the growth of surrounding vegetation, adversely impacting crops such as peanuts, sunflowers, corn, soybeans, and wheat [27]. In agricultural systems, high densities of *A. psilostachya* have been linked to notable yield reductions. Additionally, the species produces highly allergenic pollen, ranking as the second most significant allergen in the United States, affecting nearly 25% of the population [28]. The impact of pollen on public health has also been highlighted in earlier work [29]. In New Zealand, risk assessments have identified *A. psilostachya* as a high-risk invasive species, emphasizing the need for robust control strategies [30].

The management of *A. psilostachya* incorporates a combination of physical, chemical, and biological methods. Cutting and herbicide application are the most commonly used strategies in agroecosystems [31]. Chemical control has been explored using herbicides such as florasulam, prosulfuron, oxadiargyl, metribuzin, bifenox, and clopyralid; however, these have demonstrated susceptibility to dicamba and 2,4-D [32]. The use of herbicides with multiple modes of action is recommended to mitigate the development of resistance [33,34]. Previous studies revealed that *A. trifida* responds strongly to dicamba and 2,4-D, with saflufenacil causing substantial reductions in plant biomass within eight weeks of application [35]. Biological control using the beetle *Ophraella communa* (Coleoptera: *Chrysomelidae*) has shown promise against *A. artemisiifolia*, reducing plant biomass by defoliating before seed formation [36], although concerns remain regarding flower damage and reduced pollen production [37].

#### 3.1.2. *Boreava orientalis*: Jaub. & Spach

**Figure 2:**
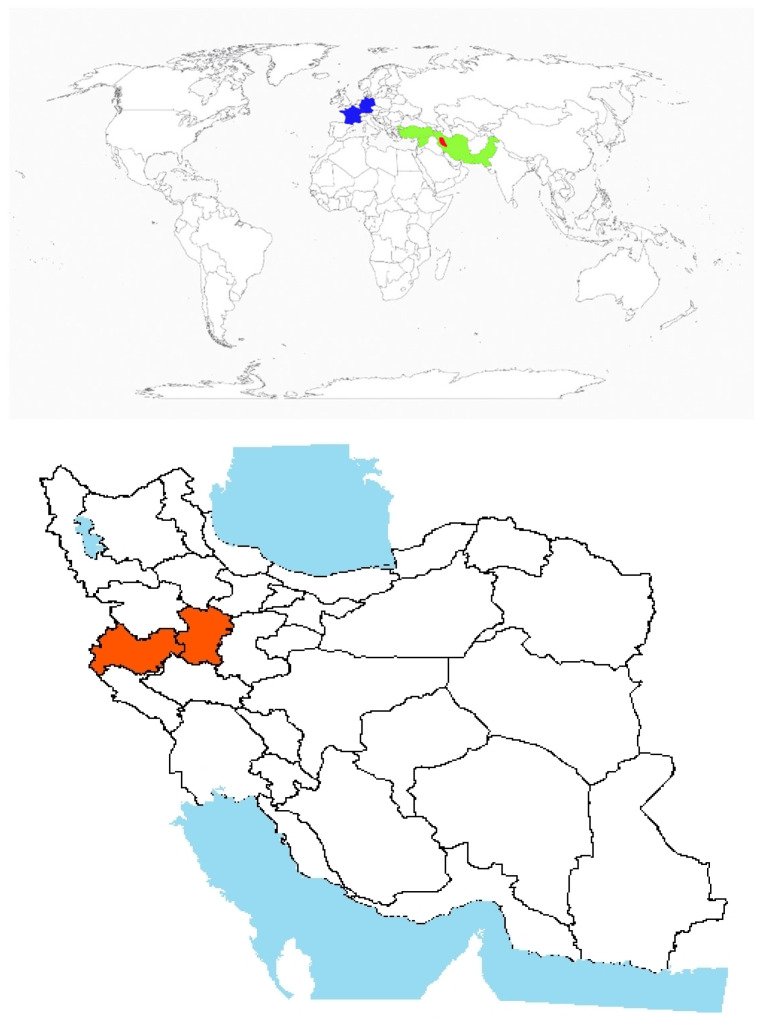
Global and national distribution of *Boreava orientalis* Jaub. & Spach. **Top panel**: Global distribution map showing native (green), invasive (red), and introduced (blue) ranges based on the literature and GBIF records. **Bottom panel:** Occurrence invasive (red) in Iran based on herbarium records, published studies, and recent field observations by the authors. Note: The absence of occurrence in some regions may reflect data gaps or lack of confirmed records and not necessarily ecological unsuitability.

- Origin and global invasiveness: It is native to parts of western Asia, including Iran, Pakistan, Syria, and Turkey (Figure 2). It is widespread from Kütahya to Istanbul in Turkey and from Armenia to the Euphrates [38]. In Iran, it was initially reported from Khorasan and Chaharmahal Bakhtiari (not as a weed), but it has been more recently identified in Kurdistan croplands [39]. According to the Manual of Alien Plants of Belgium records also document its presence in Belgium, Germany, France, the United Kingdom, and Pakistan [40].
- Morphological description, biology, and ecology: *B. orientalis* Jaub. & Spach is an annual glabrous herb that reaches 15–30 cm in height and typically branches above the middle of the plant into a loose, ebracteate inflorescence [41]. It exhibits a glaucous appearance with yellow flowers and simple leaves. Basal leaves are oblong-lanceolate, clasping, auriculate or sagittate, with entire margins and acute apices, while cauline leaves are also sagittate. The petals are approximately 5 mm long, and the calyx is open, not or scarcely saccate. Petals are spathulate to oblong–lanceolate, filaments are slightly dilated, and the ovary is 1–2-ovulate with a capitate stigma. The slender pedicels, 6–8 mm long in fruit, are erect or spreading. The fruit is indehiscent, one-seeded, beaked, ovate, and may be winged or wingless; it is 8–10 mm long, somewhat tubercled, and plicate between the wings. The radicle is incumbent, and the species has a chromosome number of 14n with small-sized chromosomes [41].

Specific climatic requirements for *B. orientalis* remain largely undocumented; however, this species is considered synonymous with *Isatis* sp., and thus likely shares similar ecological preferences. It requires vernalization for flowering, with crown and seedling rosettes exhibiting different responses to temperature variations [42]. The species appears to be adapted to environments with cold winters and warm to hot summers, consistent with habitats occupied by *Isatis* species across the Eurasian steppes, Morocco, and even Arizona. For example, *Isatis tinctoria* commonly coexists with *Artemisia tridentata*, a species capable of tolerating field temperatures ranging from −18 °C to 38 °C [43], with leaf tissue tolerances reaching up to 46 °C [44]. The dispersal of *B. orientalis* fruits typically occurs near the parent plant—within 50 cm—although greater distances can be achieved via insects, water, human and animal activity, or contaminated seed lots [45]. Seed viability in soil is relatively short-lived, with most seeds losing viability within 12 months, likely due to factors such as soil moisture content [42].

- Agricultural impact and management challenges: *B. orientalis* is a winter annual weed that commonly infests irrigated and rain-fed fields of wheat, barley, chickpeas, and rapeseed [39,46]. It is rarely found in non-arable lands such as pastures and gardens, likely due to the absence of annual tillage. This species has also been reported in winter wheat fields on the Anatolian Plateau of Turkey [47]. Notably, *B. orientalis* exhibits allelopathic activity through the release of isothiocyanates derived from glucosinolate breakdown, which may contribute to its competitiveness and render it unpalatable or toxic to livestock [48].

Despite its growing presence in crop systems, there are currently no published studies detailing specific control measures for *B. orientalis*. While it has not yet caused widespread crop damage, field evidence indicates that commonly used herbicides, such as tribenuron, have spread to livestock [48], and 2,4-D are ineffective against this species in western Iran [49,50], highlighting the need for targeted management strategies.

#### 3.1.3. *Cynanchum acutum* L.

**Figure 3:**
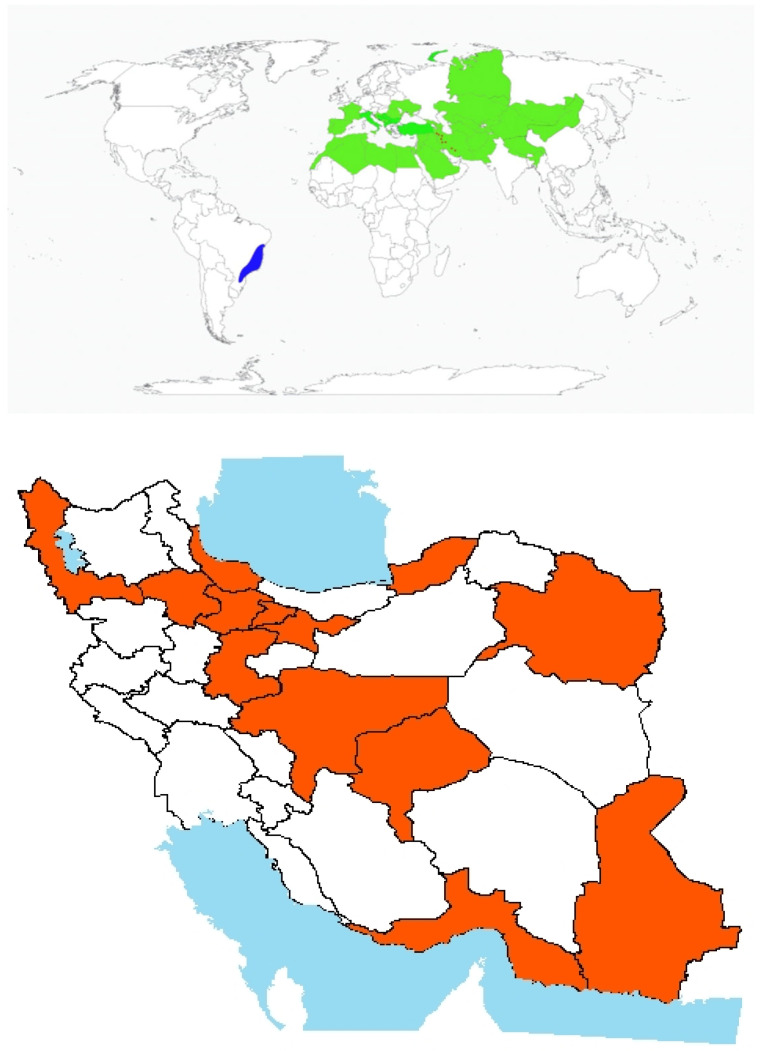
Global and national distribution of *Cynanchum acutum* L. **Top panel**: Global distribution map showing native (green), invasive (red), and introduced (blue) ranges based on the literature and GBIF records. **Bottom panel:** Occurrence invasive (red) in Iran based on herbarium records, published studies, and recent field observations by the authors. Note: The absence of occurrence in some regions may reflect data gaps or lack of confirmed records and not necessarily ecological unsuitability.

- Origin and global invasiveness: The genus name “*Cynanchum*” is derived from the Greek meaning “dog strangler”, likely referencing its twining growth habit, while “acutum” alludes to its sharply pointed leaves [51]. Its origin lies within the Mediterranean Basin, encompassing Southern Europe, North Africa, and parts of Western Asia (Figure 3) [52]. The perennial vine thrives in warm, arid climates and is frequently found in coastal regions, on sandy dunes, and in disturbed habitats [53]. In addition to its invasive potential, *Cynanchum* L. has been used in traditional medicinal practices in some cultures.

Native to Ukraine and Russia [54], *C. acutum* demonstrates a broad geographic range extending from southern Europe through southwest Asia and into North Africa (Figure 3) [55]. In Iran, the species is widespread across diverse climatic regions and is commonly found in the Iranoturani phytogeographical zone. It occurs extensively in the northern provinces (Gorgan and Guilan), as well as the northwestern, central, eastern, and southern provinces throughout the country (Figure 3). Additionally, the species has been introduced to southeastern Brazil, further illustrating its adaptive capacity to varied environmental conditions (Figure 3).

- Morphological description, biology, and ecology: *C. acutum* is a perennial species distinguished by either glabrous surfaces or multicellular hairs, exhibiting fibrous, fleshy, or woody roots with greenish stems. Its leaves are opposite, petiolate, and typically cordate, elliptical, ovate, or obovate, with entire margins and acute apices. Occasionally, small leafy stipules are present. The plant produces extra-axillary inflorescences that can be raceme-like, corymbose, or umbel-like. Flowers range in diameter from 3 to 15 mm, displaying imbricate or contorted aestivation with dextrorse orientation. They are nectariferous and have a divided corolla that is contorted in the bud stage, appearing rotate, sub-rotate, or tubular in shape. Flower coloration varies from white to green and yellow, and occasionally reddish. The calyx is free to the base, composed of erect sepals often containing basal glands. The seeds are oval, flat, and brown, well-adapted for wind dispersal, facilitating long-distance propagation [41,56].

*C. acutum* is commonly found across diverse habitats, including beaches, riverbeds, hedges, fields, gardens, roadsides, and relatively saline soils, showing a preference for moist environments [55]. It is highly adaptable to various soil types due to its phenotypic plasticity, which enables it to survive under a broad range of environmental conditions. Seed germination initiates at approximately 17 °C and reaches optimal levels at 25 °C. The species has a robust root system that penetrates at least 10 cm deep into the soil, complicating eradication efforts.

- Ecological impact and management strategies: *C. acutum* possesses a robust root system and an ascending, twining stem that wraps around tree trunks, extending into branches and obstructing light penetration to the canopy. This growth behavior damages tree buds and inhibits photosynthesis. It produces flowers and seeds on exposure to light [57]. Control methods include mechanical removal and herbicide application. This deep-rooting behavior enhances its ability to persist and regenerate, making it a challenging invasive species. Effective herbicides include glufosinate ammonium [58], paraquat, and glyphosate [56], while picloram and nicosulfuron have shown limited efficacy [59]. As such, the development of effective, long-term management strategies is essential for mitigating its ecological and agricultural impact.

#### 3.1.4. *Ibicella lutea* (Lindl.) Van Eselt.

**Figure 4:**
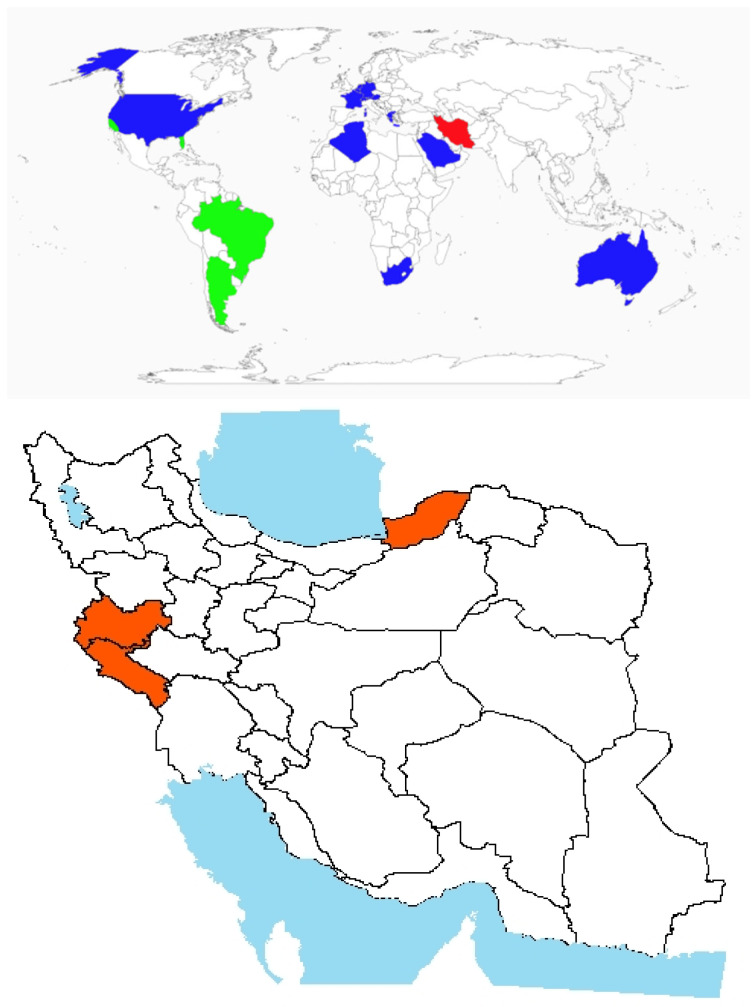
Global and national distribution of *Ibicella lutea* (Lindl.) Van Eselt. **Top panel**: Global distribution map showing native (green), invasive (red), and introduced (blue) ranges based on the literature and GBIF records. **Bottom panel:** Occurrence invasive (red) in Iran based on herbarium records, published studies, and recent field observations by the authors. Note: The absence of occurrence in some regions may reflect data gaps or lack of confirmed records and not necessarily ecological unsuitability.

- Origin and global invasiveness: The family *Martyniaceae* comprises five genera and 16 species globally, with the greatest concentration in Argentina and other parts of South America (Figure 4) [60]. A distinguishing morphological trait of this family is the presence of fruits with a curved beak, which may be longer or shorter than the fruit body [61]. The genus *Ibicella*, part of *Martyniaceae*, includes eight species that are naturally distributed from South America to Mexico [62]. *I. lutea* (Lindl.) Van Eselt., commonly known as the yellow unicorn plant, has previously been misidentified in various studies. For instance, a study on Lesos Island in the eastern Aegean region misclassified a population of *I. lutea*, later clarified to be aligned with *Proboscidea louisianica* (Mill.) Thell. ssp. *louisianica* based on Bretting’s interspecific classification [63,64]. While *I. lutea* has been recorded in neighboring countries such as Turkey, no members of the genus *Proboscidea* have yet been documented there [65].

Species within the *Martyniaceae* family demonstrate a strong ecological resilience, particularly in degraded habitats and arid to semi-arid regions. These plants are commonly found along roadsides, as well as in sandy deserts, meadows, beaches, pastures, and sand dunes, thriving up to elevations of 1800 m [63]. Native to the arid regions of Texas and Mexico, members of *Ibicella* have dispersed throughout South and North America, with introductions from the United States to other parts of the world [66]. The species was first reported in Greece, which also makes it the first record for the Eastern Mediterranean region (Figure 4) [64]. In Iran, *I. lutea* was first documented as a weed in cotton fields in the Gorgan and Gonbad regions, with its introduction speculated to have originated from Saudi Arabia [67]. Recent surveys conducted during 2018–2019 confirmed its presence in western Iran, particularly in the Ilam and Kermanshah Provinces. These included sites such as the landscape of Ilam University and a sugar beet field in Badreh Gard Salimi Village in Homeil, Eslamabad Gharb City (Figure 4). It exhibits a strong capacity to rapidly colonize disturbed habitats, particularly during warm seasons, due to its prolific seed production, efficient seed and fruit dispersal, and climatic adaptability. The absence of natural predators or competitors in newly invaded areas further facilitates its establishment. This ecological advantage contributes to its aggressive spread, posing serious challenges for summer crops and altering landscape dynamics [68].

- Morphological description, biology, and ecology: *I. lutea*, a member of the *Martyniaceae* family in the order Lamiales, is an annual plant with tuberous roots, native to the New World. The family includes both annual and perennial herbaceous plants, noted for their glandular hairs and strong fragrance [69]. *I. lutea* typically reaches heights of 20–120 cm and is characterized by hairy, nearly circular leaves and clusters of bright yellow flowers with red spots internally. The inflorescences bear long petioles that may either terminate or split, with broad, scaly bracts situated just below the flowers [64]. The flowers are bisexual, composed of five unequal sepals and a bilabiate corolla, with stamens arranged epipetally—two long, two short, and one vestigial. The superior ovary exhibits parietal placentation and terminates in a two-lobed stigma that often closes upon contact. The fruit, initially fleshy, matures into a woody capsule with a prominent beaked endocarp, culminating in two curved, horn-like projections longer than the fruit body itself [69]. The seeds are dark, flattened, and uneven in texture, while the fruit morphology varies from smooth to rough, resembling the spiny capsules of *Datura stramonium* L.

Ecologically, *Martyniaceae* plants demonstrate notable resilience, commonly found in arid and semi-arid environments. Their claw-shaped pods are well-adapted for zoochorous dispersal, attaching to the fur and hooves of large animals, which facilitates the spread of seeds across diverse habitats [70,71]. This adaptation plays a significant role in the invasive potential of *I. lutea*. Flowering typically begins by late October, and while immature fruits are present, it remains uncertain whether they can reach full maturity under agricultural conditions. The distinctive morphology of *I. lutea* fruit has also contribued to its anthropogenic dissemination, as humans have used the horn-shaped seed pods ornamentally, contributing to its unintentional spread [68]. Known commonly as “Devil’s Claw” or “Yellow Unicorn Plant”, the fruit’s bifurcated, claw-like form gives the species its colloquial name and enhances its ecological and cultural significance.

- Agricultural impact and management strategies: Field observations and research indicate that *I. lutea* poses a growing threat to both agricultural and natural ecosystems, particularly in arid and semi-arid regions across the southern United States. Its large size and vigorous growth habit have demonstrated significant competitive ability against both pasture and row crops. Studies show that *I. lutea* can reduce cotton (*Gossypium hirsutum* L.) yields by 60 to 74% [72], with weekly interference resulting in incremental yield losses of up to 5% [73]. Furthermore, its competitive effect has been observed to extend as far as 0.5 m from neighboring plants by the season’s end [72]. Although previously undocumented in Iran, recent reports confirm its invasion in the Ilam and Kermanshah Provinces, indicating a recent introduction [68].

Effective management of *I. lutea* begins with preventive strategies. Due to its invasive potential, it should be incorporated into regional floristic records. It is crucial to understand the mechanism underlying its rapid spread in *I. lutea* in the Ilam and Kermanshah Provinces during warm seasons. Immediate implementation of plant quarantine regulations and ongoing surveillance of farms and orchards are essential to contain its spread and limit its ecological and agricultural impact [68]. Moreover, this includes avoiding the use of unpacked or contaminated seed lots and refraining from applying fresh manure, which may harbor viable weed seeds. Physical exclusion methods, such as restricting livestock access to infested sites and the proper cleaning of tools, footwear, and machinery, are critical in minimizing seed dispersal. Enhancing field sanitation, such as by cementing irrigation channels and adopting pressurized irrigation systems, can also create conditions less favorable to weed establishment [74]. Mechanical control methods, including early-season cutting of plants before fruit formation, combined with crop rotation, can help suppress weed populations. Chemical control has also proven effective, with registered herbicides such as 2,4-D showing good efficacy [75]. Additionally, pre-plant herbicides like trifluralin have been successfully used in bromoxynil- and glyphosate-resistant cotton fields [76]. Eco-physiological studies are needed to assess germination response to local climatic factors, particularly its optimal germination temperature of 20 °C and its salinity tolerance threshold (~80 mmol). Field experiments must evaluate their competitive behavior in summer crops and assess landscape-level ecological impacts. These integrated strategies offer a multifaceted approach to managing *I. lutea* and safeguarding crop productivity.

#### 3.1.5. *Picnomon acarna* (L.) Cass.

**Figure 5:**
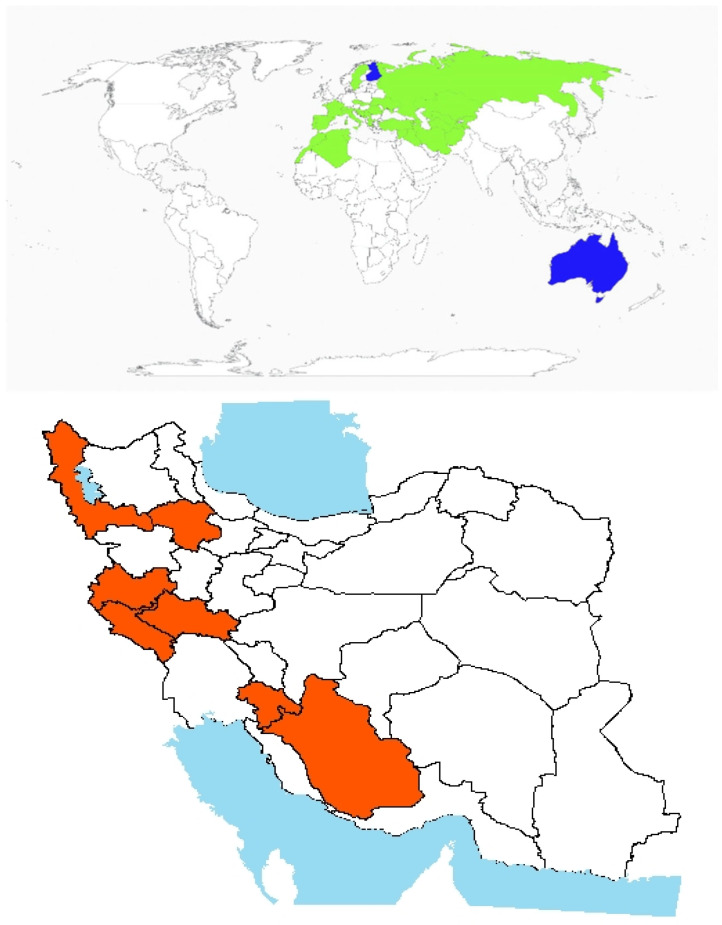
Global and national distribution of *Picnomon acarna* (L.) Cass. **Top panel**: Global distribution map showing native (green), and introduced (blue) ranges based on the literature and GBIF records. **Bottom panel:** Occurrence invasive (red) in Iran based on herbarium records, published studies, and recent field observations by the authors. Note: The absence of occurrence in some regions may reflect data gaps or lack of confirmed records and not necessarily ecological unsuitability.

- Origin and global invasiveness: *P. acarna* (L.) Cass. is a spiny, annual, and herbaceous plant native to the Mediterranean region (Figure 5). Initially prevalent in neglected areas and rangelands, *P. acarna* has become a major invasive weed in rainfed agricultural fields, especially in western Iran [77]. It has also been reported in the western regions of Victoria and the southeastern parts of South Australia (Figure 5). The species’ non-palatability and resilience to soil compaction and erosion have enabled its proliferation in Iranian forests and pastures (Figure 5) [78]. The increasing adoption of conservation tillage and reduced soil disturbance in rainfed systems [79] has further facilitated the spread of *P. acarna*.

- Morphological description, biology, and ecology: Belonging to the Asteraceae family, it exhibits typical morphological traits such as spiny, hairy leaves that can accumulate dust and reduce herbicide absorption [22]. A notable characteristic of this plant is its height, reaching approximately 50 cm, with leaves bearing spines that range from 10 to 15 cm in length [80]. The species produces seeds with a pappus measuring 1 to 2 cm in length, aiding in wind dispersal over long distances [81]. Notably, its seeds are primarily photoblastic, meaning that they require light for germination [82]. This germination strategy contributes to its success in open and disturbed habitats. Its effective seed dispersal and environmental adaptability enhance its invasive potential across diverse habitats [82].
- Agricultural impact and management strategies: The proliferation of *P. acarna* has intensified in rainfed agricultural systems due to favorable environmental and management conditions [83]. This invasive species poses a significant threat to key rainfed crops, such as wheat (*Triticum aestivum* L.) and chickpeas (*Cicer arietinum* L.). Its rigid, spinous structure severely complicates harvest operations, particularly for crops like chickpeas and lentils, which are traditionally harvested by hand. In fields heavily infested with *P. acarna*, farmers often abandon cultivation altogether due to the high risk of injury posed by the weed’s long spines [84]. Up to 25% yield reduction can occur at a density of 16 plants of *P. acarna* per square meter [82].

The seeds of *P. acarna* are photoblastic, requiring light for germination, which presents challenges for tillage-based management strategies. Tillage operations conducted in early spring, when soil moisture is sufficient, may inadvertently promote emergence by exposing buried seeds to light [82]. An effective cultural strategy involves inter-row cultivation during the warmer parts of the growing season, particularly in legume crops. This approach helps bury seeds deeper in the soil, reducing light exposure and thereby inhibiting germination [85]. In cereal crops such as wheat and barley, increasing crop density has also shown promise in suppressing *P. acarna* by shading the soil surface and limiting the light availability necessary for seed germination, ultimately reducing weed establishment and competition. Although herbicides have proven effective in managing *P. acarna*, their application remains limited in Iranian rainfed crops, including wheat, barley, chickpeas, and lentils [83].

#### 3.1.6. *Physalis divaricata* D. Don

**Figure 6:**
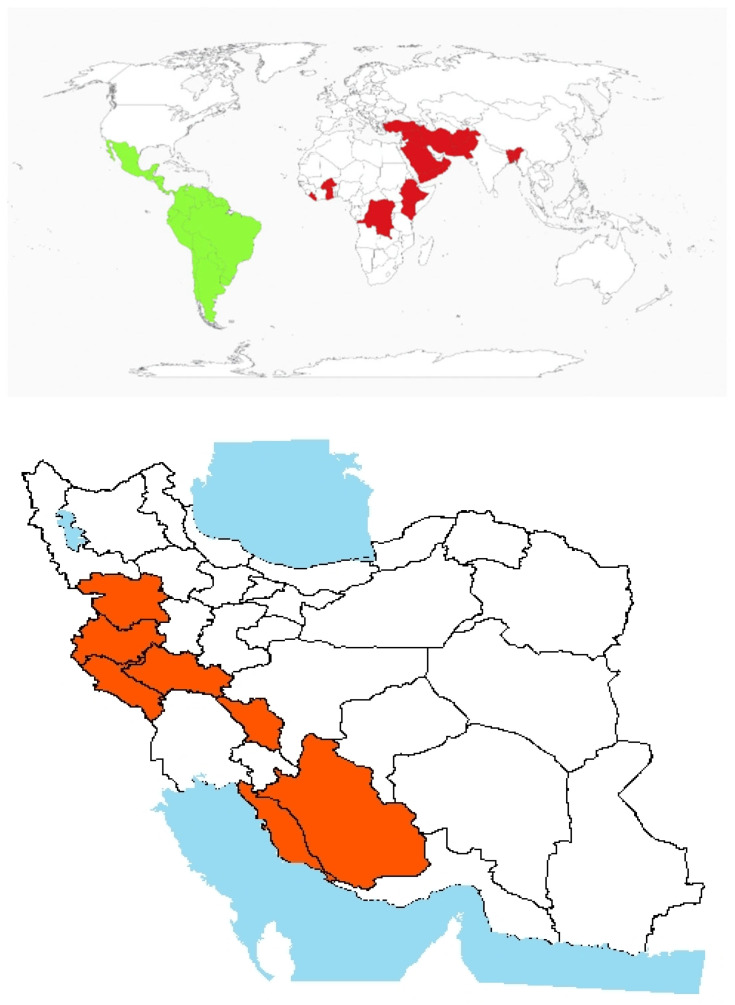
Global and national distribution of *Physalis divaricata* D. Don. **Top panel**: Global distribution map showing native (green) and invasive (red) ranges based on the literature and GBIF records. **Bottom panel:** Occurrence invasive (red) in Iran based on herbarium records, published studies, and recent field observations by the authors. Note: The absence of occurrence in some regions may reflect data gaps or lack of confirmed records and not necessarily ecological unsuitability.

- Origin and global invasiveness: *P. divaricata* D. Don, commonly known as Annual Groundcherry, is primarily native to Latin America and South America (Figure 6). Vargas et al. (2001) classify it as native to Mexico, while Ramírez and Davenport (2016) recognize Colombia as a major centre of its natural distribution [86,87].

This species has spread widely across parts of the Middle East and South Asia, including Afghanistan, Pakistan, Turkey, Iraq, Syria, Saudi Arabia, Kuwait, Oman, and Yemen [52]. It was introduced into Iran in 1984, with its first recorded presence in Fars Province [67]. The annual species *P. divaricata* D. Don is the most prevalent among *Physalis* species in Iran and has become well-established in crop fields, particularly in western regions. It is also recognized as toxic [88], posing potential risks to agricultural and ecological systems (Figure 6). Similar to *I. lutea*, its invasive behavior is also driven by its rapid colonization of disturbed habitats during warm seasons. Its success is attributed to prolific seed production, efficient fruit and seed dispersal, and climatic adaptability. The absence of natural enemies and lack of evolutionary coexistence in newly invaded regions further enhances its establishment across diverse ecosystems, promoting persistent weed infestation.

- Morphological description, biology and ecology: The *Solanaceae* family includes approximately 2500 species across 90 genera, predominantly found in warm regions such as Central and South America, and temperate climates worldwide. This diverse plant family ranges from herbaceous annuals to woody perennials. Their leaves are typically alternate with variable shapes and sizes, while the flowers are mostly unisexual and occasionally hermaphroditic. Floral structures usually exhibit pentamerous symmetry, although variability exists. Fruits are commonly berries or capsules [67]. As a member of this family, it is an annual plant that produces berry-type fruits which transition in color from green to purple black upon ripening. The immature berries are known to be toxic [89]. Fruiting generally occurs by late June, with seed germination peaking approximately four weeks later. Seed viability remains high when buried at depths of 10–30 cm, whereas surface-level seeds are more susceptible to desiccation and mortality. Germination typically begins in March following winter moisture absorption in January, with full fruit development occurring by April [90]. The seeds can exhibit a germination rate of 93% under alternating temperatures of 10/20 °C (night/day), although germination drops by 41% under dark conditions at the same temperature. Salinity also affects germination: a concentration of 22.85 mM reduces the maximum germination by 50%. Additionally, soil pH levels between 6 and 7 are optimal for germination, indicating the species’ sensitivity to environmental factors such as temperature, light, salinity, and acidity [91].

Ecologically, *P. divaricata* demonstrates morphological plasticity and thrives under varying environmental conditions. Despite its widespread occurrence, it remains under-researched. The extent of its impact is influenced by factors such as the timing of weed emergence, crop type, competition from other weeds, and crop density [92].

- Agricultural impact and management strategies: *P. divaricata* is recognized as a problematic weed across various agricultural systems in Iran due to its entangling growth habit, which complicates mechanical harvesting, particularly in fine grain and sugar beet crops [90,93]. In potato fields and sugar beet systems, its synchronous growth cycle exacerbates competition with crops [94,95]. Its competitive presence is most pronounced during the summer months in summer crops across the Lorestan and Chaharmahal Bakhtiari Provinces [94]. The weed has also been reported in orchards in Lorestan [94] and Dare Shahr in Ilam Province [68]. Notably, high-density infestations of *P. divaricata* can lead to crop yield reductions of 50–60% [96]. Additionally, *P. divaricata* has been observed in tomato fields in southeastern Iran [97], highlighting its broad ecological range and significant agricultural impact.

The effective management of *P. divaricata* requires an integrated approach that combines preventive, chemical, cultural, and biological strategies to reduce weed density, limit its spread, and minimize control costs. By implementing these measures, farmers can effectively manage weeds, reduce control costs, and maintain weed-free fields, ultimately enhancing agricultural productivity [74]. Regular field monitoring is essential for assessing weed populations and guiding timely interventions. Preventive measures such as avoiding the use of unpacked or contaminated seeds and refraining from applying fresh or decomposed manure are critical in reducing initial seed introductions into agricultural fields [74]. Weed emergence around irrigation streams and field margins can be suppressed by cementing main water channels and utilizing pressurized irrigation systems, which create less favorable environments for weed establishment.

Cultural control practices, particularly crop rotation, have also proven effective. Rotating with wheat significantly reduced the emergence and biomass of *P. divaricata*, offering a practical approach to long-term weed suppression [90]. Biological control is another emerging area of interest. Isolate 6A of *Alternaria alternata* has demonstrated pathogenic effects on *P. divaricata*, particularly at the six-leaf stage, when applied at a concentration of 10^8^ spores mL^−1^. This highlights the potential of *A. alternata* as a biological agent in integrated weed management programs [98]. Further research is required to optimize application methods, dosages, and assess non-target impacts on other flora and soil biota.

Chemical control remains an essential component of *P. divaricata* management. The application of contact herbicides, like paraquat, or systemic options, such as glyphosate, around canals and ditches has proven effective in reducing weed infestations. Among pre-emergent herbicides, chloridazone has demonstrated significant efficacy, inhibiting over 50% of seed germination in treated soils compared to the untreated control at a 5% significance level [99]. Three weeks after application, all seedlings exposed to chloridazone were destroyed, while those in the control plots remained unaffected. In contrast, herbicides such as trifluralin and ethalfluralin, whether applied alone or in combination, did not significantly reduce germination rates [99]. Additionally, foramsulfuron and nicosulfuron at 56 g ai ha^−1^ in corn fields, and phenmedipham at 547 g ai ha^−1^ in sugar beet fields, have shown promising results in reducing weed density and growth [100].

#### 3.1.7. *Vicia hyrcanica* Fisch. & C.A. Mey.

**Figure 7:**
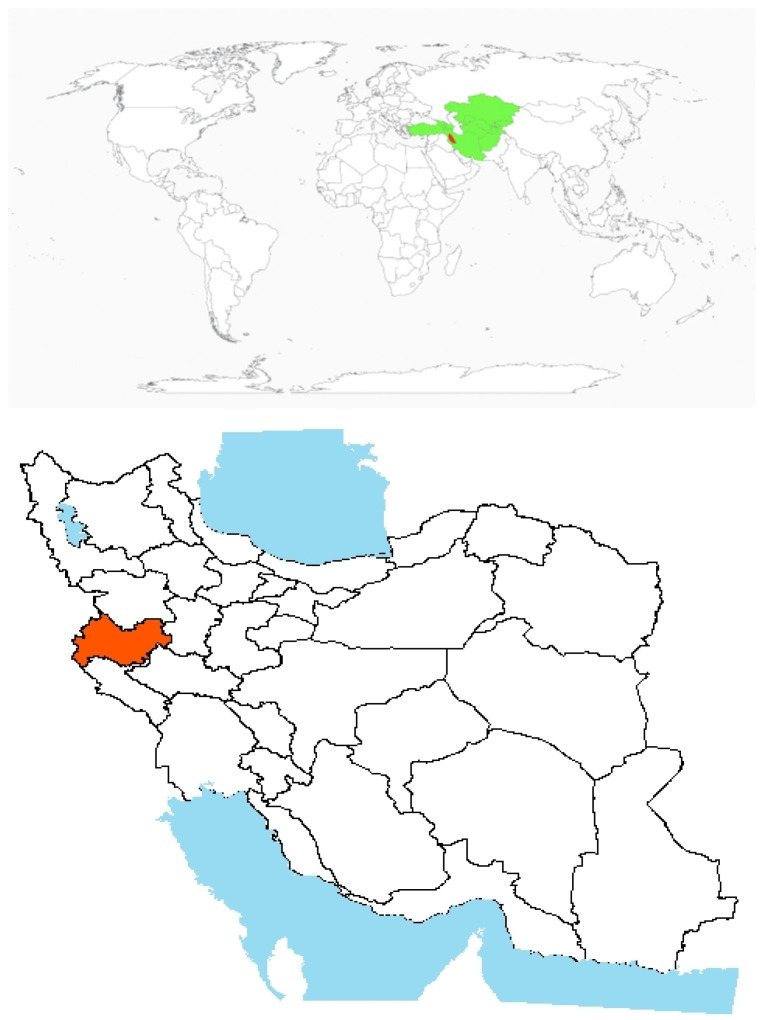
Global and national distribution of *Vicia hyrcanica* Fisch. & C.A. Mey. **Top panel**: Global distribution map showing native (green) and invasive (red), ranges based on the literature and GBIF records. **Bottom panel:** Occurrence invasive (red) in Iran based on herbarium records, published studies, and recent field observations by the authors. Note: The absence of occurrence in some regions may reflect data gaps or lack of confirmed records and not necessarily ecological unsuitability.

- Origin and global invasiveness: *V. hyrcanica* Fisch. & C.A. Mey. is a climbing annual winter legume characterized by weak stems and seed-based dispersal. It is native to northern Iran and has progressively extended its range into western parts of the country [80]. The genus *Vicia* is made up of herbaceous legumes, primarily distributed across temperate regions, particularly in Mediterranean areas (Figure 7) [101,102]. Despite their wide distribution, comprehensive information on the *Vicia* genus’s natural distribution, taxonomy, and agricultural potential remains underexplored. In Iran, *V. hyrcanica* is commonly found in rainfed fields, pastures, and perennial horticultural systems. In recent years, the species has rapidly spread into both disturbed and undisturbed habitats across the country (Figure 7).

- Morphological description, biology, and ecology: *V. hyrcanica* typically germinates in late winter, aligning its life cycle with that of cultivated legume crops in Iran. The stem is erect and can reach a length of up to 80 cm. Its leaves consist of three to eight pairs of linear to narrowly elliptic leaflets, each measuring between 1.5 and 3.0 cm. The species produces solitary, yellow flowers that range from 1.0 to 1.8 cm in length. The mature legume is circular in cross-section, approximately 4 cm long, and dark brown to black in color. Upon ripening, the pod dehisces into two twisted halves, facilitating seed dispersal [103].

A notable trait of *V. hyrcanica* is its relatively large seeds, which possess a hard seed coat. This physical dormancy often delays germination following seed dispersal, contributing to its persistence and invasive behavior [104]. The scarification of its hard seed coat, promoted by cold temperatures and soil tillage, enhances germination rates. Therefore, adopting no-tillage practices may serve as a viable strategy to suppress its emergence. The species is particularly common in low-input agricultural systems and demonstrates strong tolerance to water stress. This trait enables *V. hyrcanica* to outcompete crops in drought-prone conditions [105].

- Agricultural impact and management strategies: *V. hyrcanica* is widely recognized as an important cover crop species in Iran [106]. Despite efforts in domestication and breeding, many cover crops, including *V. hyrcanica*, retain seed dormancy mechanisms inherited from their wild ancestors [107,108]. These dormancy traits can limit their effective integration into cropping systems and raise concerns about their potential weediness. The presence of seed dormancy allows *V. hyrcanica* to persist in the soil seed bank and germinate unpredictably, potentially disrupting future crop cycles. Studies have shown that both genotype and environmental factors during seed development, as well as postharvest storage conditions, influence dormancy levels and germination timing [108,109,110]. Additionally, climatic conditions during the parental plant’s development significantly impact seed germination potential [111], increasing the species’ risk of becoming invasive in new areas.

This increasing prevalence has raised environmental concerns and emphasized the need for management interventions. In cereal crops such as wheat and barley, a broad spectrum of herbicides has been reported to effectively control *V. hyrcanica*. Species such as *V. villosa* (hairy vetch), which share morphological seed characteristics with *V. hyrcanica*, are particularly difficult to manage in rainfed legumes like chickpeas and lentils due to the difficulty in distinguishing their seeds during cleaning and processing. This similarity complicates weed control and contributes to the proliferation of *V. hyrcanica* populations in legume-based cropping systems [112]. Effective management requires both chemical and cultural strategies tailored to crop type and environmental conditions.

### 3.2. Aquatic Invasive Weeds

#### 3.2.1. *Azolla filiculoides* Lam. and *Azolla pinnata*

**Figure 8:**
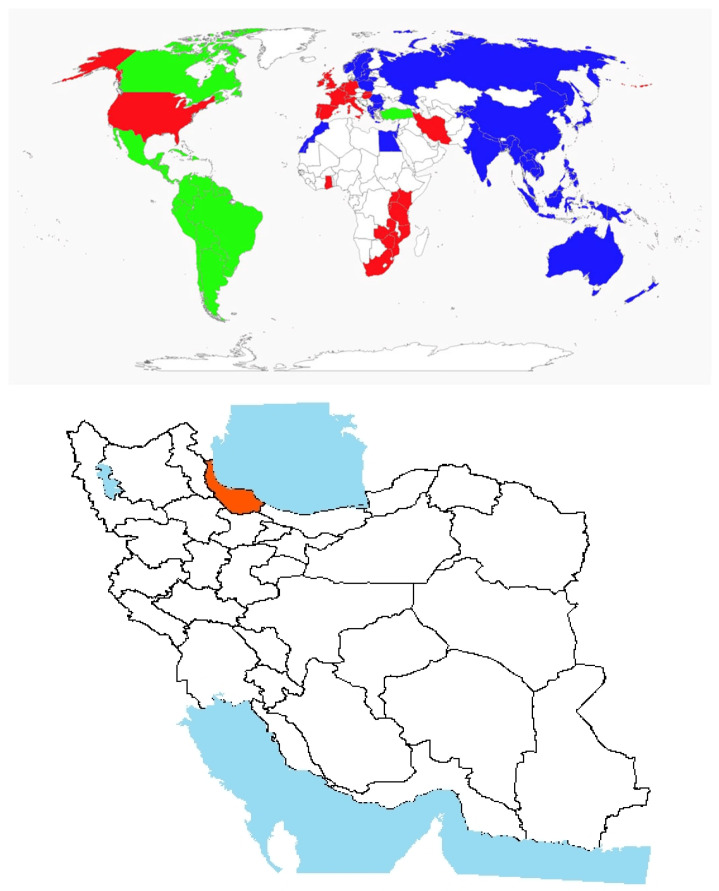
Global and national distribution of *Azolla* spp. **Top panel**: Global distribution map showing native (green), invasive (red), and introduced (blue) ranges based on the literature and GBIF records. **Bottom panel**: Occurrence invasive (red) in Iran based on herbarium records, published studies, and recent field observations by the authors. Note: The absence of occurrence in some regions may reflect data gaps or lack of confirmed records and not necessarily ecological unsuitability.

- Origin and global invasiveness: *Azolla* spp., commonly referred to as mosquitofern, represent a genus of small aquatic ferns with a rich evolutionary history. The first recorded collection of *Azolla* was made by Baptiste-Jean Lamarck in South America, although genetic differentiation among species indicates varied origins [113]. Fossil evidence suggests that *Azolla* has existed for over 70 million years, tracing its origins back to the early Cenozoic era [114]. Historically believed to have originated in the Americas, subsequent genetic studies have revealed a more complex and widespread origin. Today, *Azolla* species are recognized as having diverse evolutionary roots across tropical, subtropical, and warm temperate regions of Africa, Asia, and the Americas (Figure 8) [115], with some species naturally occurring in parts of Europe and Asia [116]. Due to its rapid vegetative growth and nitrogen-fixing ability, *Azolla* has been introduced to several regions, including Australia and parts of Africa, for agricultural and ecological purposes. However, its widespread introduction has led to invasiveness in multiple countries, such as the United States, the United Kingdom, France, Iran, and South Africa (Figure 8).
- Morphological description, biology, and ecology: *Azolla* spp. (*Salviniaceae*) are small, floating aquatic ferns characterized by polygonal or triangular shapes [117]. Most species measure between 3 and 4 cm, except for *Azolla nilotica*, which may exceed this size. The stems are covered with minute, alternate, scaly leaves arranged in two overlapping ranks. Roots are unbranched but bear fine lateral rootlets that give a feathery appearance when submerged [118]. Color and size can vary significantly depending on environmental conditions, such as light and nutrient availability [119]. *Azolla* spp. reproduce both vegetatively and sexually. While vegetative propagation is the predominant method [120], sexual reproduction occurs, involving the formation of megaspores and microspores; however, it is considered a rare phenomenon in natural populations [113].

This broad phylogeographic distribution likely reflects the genus’s ability to disperse naturally over millennia, facilitated by its capacity to adapt to a range of aquatic environments. *Azolla* spp. are commonly found in freshwater bodies, such as pits, pools, lakes, and rivers, in temperate and tropical regions. Their growth is closely linked to factors including air temperature, humidity, light intensity, and the availability of nitrates and phosphates [121]. Optimal growth occurs at 20–30 °C [122]. Humidity levels below 60% cause desiccation [123], and the preferred pH range is between 4.5 and 7 [124]. The genus plays a significant ecological role, particularly in nitrogen fixation, through its symbiotic relationship with the cyanobacterium *Anabaena azollae*, contributing to soil fertility in rice paddies and wetland systems. It forms dense mats on water surfaces, which can inhibit light penetration, reduce oxygen levels, and outcompete native aquatic vegetation, thereby disrupting aquatic ecosystems. Despite its utility in biofertilization and wastewater treatment, the unregulated proliferation of *Azolla* spp. necessitates targeted management strategies to mitigate its environmental impacts [120].

- Ecological impact and management challenges: *Azolla* spp. can severely disrupt aquatic ecosystems by reducing oxygen levels, altering water acidity, limiting light penetration, and disturbing aquatic food webs [125]. In northern Iran, particularly in the Guilan and Mazandaran Provinces, the dense proliferation of *Azolla* in Anzali Wetland has led to increased toxicity and mortality of fish and other aquatic organisms, pushing the ecosystem toward collapse [126] (Figure 8). In rice paddies, the rapid surface growth of *Azolla* forms a thick mat that interferes with rice transplanting by bending and submerging seedlings, thus impeding early crop establishment [126].

Management strategies for *Azolla* spp. include mechanical removal and chemical herbicides such as paraquat, Dicot [127], and glyphosate [128]. However, due to their environmental risks, chemical methods are generally not recommended. Also, conventional methods like chemical and mechanical control are often costly and impractical in such ecosystems. Among biological approaches, the use of the beetle *Stenopelmus* (Coleoptera: *Erirhinidae*) has proven to be the most effective and environmentally sustainable method for controlling *Azolla* spp. populations globally [129]. Research should prioritize the optimization of biological control strategies, especially in sensitive and economically constrained regions such as the Anzali Wetland in Guilan Province, Iran. Investigations into potential shifts in host specificity are also critical to ensure the safety of non-target species. Comprehensive risk assessments and long-term ecological monitoring must accompany the implementation of biological control to ensure environmental compatibility and long-term sustainability.

#### 3.2.2. *Eichhornia crassipes* (Mart.) Solms

**Figure 9:**
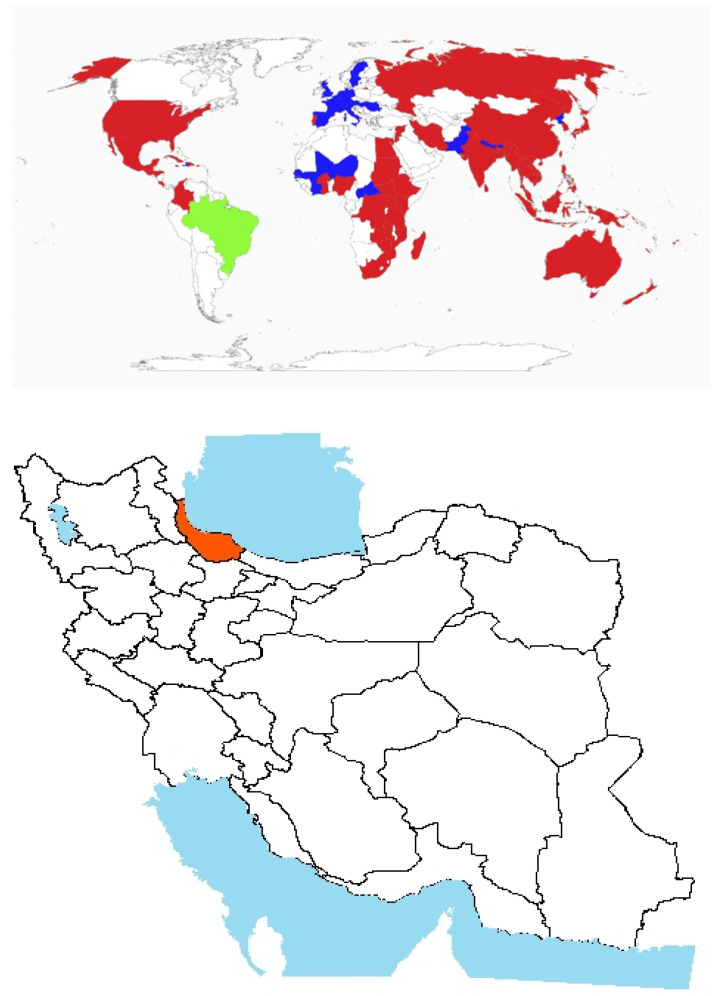
Global and national distribution of *Eichhornia crassipes* (Mart.) Solms. **Top panel**: Global distribution map showing native (green), invasive (red), and introduced (blue) ranges based on the literature and GBIF records. **Bottom panel**: Occurrence invasive (red) in Iran based on herbarium records, published studies, and recent field observations by the authors. Note: The absence of occurrence in some regions may reflect data gaps or lack of confirmed records and not necessarily ecological unsuitability.

- Origin and global invasiveness: *E. crassipes* (Mart.) Solms (Family: Pontederiaceae), commonly known as water hyacinth, is a floating aquatic plant native to the Amazon Basin in South America, where it naturally inhabits rivers, lakes, and wetlands (Figure 9) [130]. It is well adapted to tropical and subtropical climates, thriving in nutrient-rich freshwater environments. The species was first described in 1842 by the German botanist Kunth. Initially introduced outside its native range as an ornamental plant in the late 19th and early 20th centuries, *E. crassipes* quickly gained popularity due to its attractive appearance. However, its ability to reproduce rapidly through both vegetative and sexual means has made it one of the most invasive aquatic plants globally [131].

Following its introduction, *E. crassipes* spread aggressively across multiple continents, particularly impacting Asia by the 1940s, with notable infestations in Japan and Indonesia [132]. Today, it is reported in over 50 countries, including regions in Africa, Asia, Australia, and North America (Figure 9) [133]. Severe infestations have been documented in Africa, India, and Spain, where the plant has disrupted aquatic systems and local economies [134]. In Iran, *E. crassipes* was first observed in the Ainak Wetland near Rasht, Guilan Province. Its rapid growth led to a significant expansion from one hectare in 2011 to 600 hectares by 2015 [135], and to 800 hectares by 2019, according to the Iran Environment Center. Although high salinity along the southern Caspian coast limits its spread, the favorable climate and abundant freshwater in northern Iran continue to support its proliferation (Figure 9). Prompt management efforts are essential to mitigate their growing environmental and economic impacts.

- Morphological description, biology, and ecology: The plant is characterized by its circular to oval-shaped leaves with flexible, spongy-covered petioles and lilac to blue flowers. The roots are fibrous and unbranched [136], facilitating nutrient absorption while minimizing resistance in aquatic conditions. Mature plants exhibit pendant roots, rhizomes, stolons, leaves, inflorescences, and fruit. The air-filled sacs in their leaves and stems allow it to remain on the water’s surface [137].

*E. crassipes* demonstrates rapid growth, with populations capable of doubling within two weeks in favorable aquatic environments. Its development is influenced by nutrient levels, temperature, salinity, herbivory, and pathogens [138]. The growth of *E. crassipes* ceases when water temperatures exceed 40 °C or drop below 10 °C, with leaves particularly susceptible to damage in frosty conditions [139]. A decline in its spread during 2016 was linked to a preceding cold winter [140]. Elevated salinity and electrical conductivity (EC) have also been shown to limit habitat suitability, restricting the plant’s distribution and survival [141,142].

- Ecological impact and management challenges: *E. crassipes* poses severe threats to aquatic ecosystems by reducing water pH and oxygen levels, obstructing waterways, accelerating sedimentation [143], and disrupting native biodiversity and activities such as fishing, irrigation, and transportation [142]. Its dense mats significantly hinder light penetration, degrading water quality and aquatic health, which, in turn, threatens food security [144]. Furthermore, it depletes essential nutrients in water bodies [145], contributing to the ecological decline of wetlands. The unchecked proliferation of *E. crassipes* often leads to habitat monopolization, outcompeting native flora and fauna [146]. In some wetlands, the increased biomass of this invasive species has resulted in ecosystem collapse. The economic burden of managing *E. crassipes* is substantial, with global control efforts costing an estimated USD 124 million annually [147]. Despite its invasive nature, it has also been investigated for beneficial applications such as phytoremediation and bioenergy production [148].

Due to its aggressive growth and resilience, *E. crassipes* remains difficult to control, necessitating a combination of management techniques [149]. Physical control, including manual and mechanical removal, is effective for small-scale infestations in shallow or confined water bodies. Biological control options, using specific insects and fungi, offer environmentally sustainable alternatives. Chemical control remains one of the most common large-scale strategies; however, concerns over environmental toxicity have driven research into safer systemic herbicides [150,151]. Herbicides like 2,4-D and bispyribac-sodium have demonstrated effectiveness in controlling *E. crassipes* [152,153,154]. In Iran, glyphosate and 2,4-D have been successfully applied to reduce its spread and mitigate associated ecological and economic damages [155]. Integrated management combining these approaches is essential for long-term control.

## 4. Policy and Stakeholder Engagement

The effective management of invasive weed species in Iran requires a collaborative framework that involves local farmers, researchers, and policymakers. Farmers, as frontline observers, play a critical role in the early detection of invasive species; however, their ability to respond is often constrained by inadequate awareness and limited access to control resources [156,157]. Enhancing public education through targeted awareness campaigns and integrating traditional ecological knowledge with scientific practices can strengthen community participation in management strategies [158,159]. Policymakers are responsible for formulating and enforcing regulations. Yet, challenges such as regulatory fragmentation and insufficient inter-agency coordination impede the comprehensive management of invasive species [157,160]. The absence of specific legislation targeting invasive weeds becomes a significant policy gap. Moreover, public involvement in policy design is minimal, further limiting efficacy. Research efforts, while essential, are hindered by insufficient baseline data and restricted funding.

## 5. Conclusions

This review underscores the alarming proliferation of invasive weed species across Iran’s agroecosystems, rangelands, and aquatic environments. Key invaders such as *B. orientalis*, *I. lutea*, *P. divaricata*, *Azolla* spp., *E. crassipes*, and *A. psilostachya* exhibit high phenotypic plasticity, robust reproductive strategies, and resistance to common control measures. Their ecological traits, including allelopathy, dormancy, and aggressive vegetative spread, enable them to displace native flora, alter ecosystem dynamics, and reduce agricultural productivity. The review highlights a critical gap in ecological data for many species, hindering the development of effective, species-specific management protocols. Current management strategies remain fragmented and emphasize the urgent need for multi-tiered approaches that combine various control methods, technological innovation, and stakeholder engagement. Invasive weeds in Iran are not merely a national concern but reflect broader global trends driven by climate change and globalization. This synthesis serves as a foundation for targeted research and coordinated management, reinforcing the necessity of proactive, interdisciplinary efforts to mitigate further degradation of Iran’s ecosystems and agricultural resilience.

## 6. Future Research Direction

The future management of invasive plant species requires the integration of advanced technologies, ecological understanding, and collaborative frameworks. Effective mitigation requires integrated approaches that consider the species-specific and ecosystem-specific dynamics. Continued scientific investigation, policy development, and community engagement are essential for minimizing the ecological and agricultural impacts of invasive plants and preserving biodiversity and ecosystem resilience.

1. Research should prioritize the development of herbicide formulations specifically tailored to the physiological and ecological traits of invasive species.
2. The introduction of non-native plants such as *Azolla* spp., *I. lutea*, and *P. acarna* into new habitats, including Iran, underscores the urgent need for comprehensive risk assessments and monitoring systems. Remote sensing and GIS technologies can significantly enhance early detection, spatial analysis, and targeted control strategies.
3. For managing *E. crassipes*, studies should investigate the biotic and abiotic factors in its native range that naturally regulate its growth to inform new management tactics.
4. Research should focus on developing tillage methods, particularly nocturnal tillage, to inhibit *P. acarna* seed germination. As its photoblastic seeds require light, nighttime soil disturbance may prevent emergence by keeping seeds in darkness. Studies must identify optimal tillage depth, timing, and frequency, while assessing impacts on soil health, biodiversity, and crop yield.
5. Future research should focus on the weed’s germination biology under various environmental conditions. Germination rates of 93% of *P. divaricata* at 10/20 °C (night/day) and the 50% reduction under salinity levels of 22.85 mM indicate that environmental manipulation could be leveraged for control.
6. For *B. orientalis*, there is a need to assess the effectiveness of shifting from post-emergence to pre-emergence herbicide strategies. Field trials across diverse agro-ecological zones are required to evaluate the efficacy of pre-emergent applications in suppressing germination and reducing the risk of resistance.
7. Managing invasive species like *B. orientalis* and *A. psilostachya* calls for coordinated action involving adaptive management, public awareness, and continuous monitoring. While chemical control remains common, biological methods, such as the use of *O. communa* against *A. psilostachya*, offer sustainable alternatives.

## 7. Methodology

For this review, we gathered information on invasive plant species from a comprehensive selection of scientific sources, including peer-reviewed journal articles, books, and reputable websites. This review does not attempt to provide a complete inventory of all invasive alien plant species in Iran. Instead, it concentrates on the nine most problematic and emerging invasive weeds identified through national surveys, the peer-reviewed literature, and expert consultations. This targeted selection enables detailed coverage of each species’ classification, origin, life form, ecological and economic impacts, and management strategies.

In this study the nine most problematic weeds species were selected based on (i) documented invasive traits, such as rapid growth, prolific reproduction, seed dormancy, allelopathy, and broad ecological tolerance; (ii) evidence of rapid spread in Iranian agroecosystems or potential to become major threats; (iii) significant ecological or economic impacts reported in national weed surveys and peer-reviewed studies; and (iv) expert consultations with Iranian weed scientists to validate species relevance and priority.

Data collection focused on ecology, biology, distribution, and control strategies, ensuring that only reliable and scientifically validated references were used. Sources for global and national distribution included the Global Biodiversity Information Facility (GBIF), herbarium records from national and regional collections, published distribution reports in the scientific literature, data from national plant protection organizations, and recent field surveys conducted by the authors between 2018 and 2024.

All occurrence records were cross-checked to remove duplicates or questionable entries. The final validated dataset was mapped using ArcMap, Version 10.5.1, a geographic information system (GIS) software, to visualize the native, invasive, and introduced ranges of each species. This methodological approach ensured an accurate synthesis of existing knowledge and provided a robust foundation for assessing the spread and impact of these invasive plants.

## Figures and Tables

**Table 1:** Major invasive weed species in Iran categorized by ecological habitat and origin status (alien or native invasive).

Ecological Habitats	Scientific Name	Family	Common Name	Synonym	Origin	Life Cycle	Weed Status in Iran
Agricultural field weeds	*Ambrosia psilostachya* DC.	*Asteraceae*	Cuman ragweed	*Ambrosia californica* Rydb.; *Ambrosia coronopifolia* Torr. & A. Gray; *Ambrosia cumanensis* auct. non Kunth; *Ambrosia rugelii* Rydb.	Mexico	Annual Perennial	Serious/Principal Weed
	*Boreava orientalis* Jaub. & Spach	*Brassicaceae*	Waxy leaved mustard or yellow weed	*Isatis quadrivalvis*	Western Asia	Annual	Serious/Principal Weed
	*Cynanchum acutum* L.	*Asclepiadaceae*	Swallowwort or climbing milkweed	*Solenostemma acutum* (L.) Wehmer; *Vincetoxicum acutum* (L.) Kuntze	Mediterranean region	Perennial	Common Weed
	*Ibicella lutea* (Lindl.) Van Eselt.	*Martyniaceae*	Devil’s Claw	*Martynia lutea* Lindl.; *Proboscidea lutea* (Lindl.) Stapf.	Argentina and South America	Annual	Occasional/Emerging Weed
	*Picnomon acarna* (L.) Cass.	*Asteraceae*	Soldier thistle	*Cirsium acarna* (L.) Moench. *Carduus acarna* L.	Mediterranean region	Annual	Common Weed
	*Physalis divaricata* D. Don	*Solanaceae*	Annual Groundcherry	*Physalis halicacabum*	Latin America and South America	Annual	Common Weed
	*Vicia hyrcanica* Fisch. & C.A. Mey.	*Fabaceae*	Hyrcan vetch	*Hypechusa hyrcanica* (Fisch. & C.A.Mey.) Alef.	Mediterranean region	Annual	Occasional/Emerging Weed
Aquatic Invasive weeds	*Azolla filiculoides* Lam.	*Salviniaceae*	Pacific Mosquitofern	-	Africa, Asia, and America	Annual	Serious/Principal Weed
	*Eichhornia crassipes* (Mart.) Solms	*Pontederiaceae*	Water hyacinth	*Eichhornia speciosa* Kunth; *Piaropus crassipes* (Mart.) Raf.	Amazon Basin in South America	Perennial	Serious/Principal Weed

## Data Availability

The original contributions presented in this study are included in the article. Further inquiries can be directed to the corresponding author.
